# Ilizarov’s method for treatment of nonunion of diaphyseal fractures of the humerus

**DOI:** 10.4103/0019-5413.69319

**Published:** 2010

**Authors:** Manish Kiran, Rabi Jee

**Affiliations:** Department of Orthopaedics, SCB Medical College, Cuttack, India

**Keywords:** Non union humerus, Ilizarov’s ring fixator, bone healing

## Abstract

**Background:**

Nonunion in diaphyseal fractures of the humerus can be treated by various modalities like plating and bone grafting, exchange nailing, fibular strut grafting and Ilizarov’s method of ring fixation. To achieve union in infected nonunion in which multiple surgeries have already been done is further challenging. We conducted a prospective study wherein the outcome of the treatment of nonunion of diaphyseal fractures of the humerus by Ilizarov’s method was analyzed.

**Materials and Methods:**

Nineteen patients with diaphyseal nonunion of the humerus were treated by Ilizarov’s external fixator. These included nonunion after plating (*n*=11), intramedullary nailing (*n*=1) or conservative methods (*n*=7). In post-surgical infected nonunion (*n*=6), the implants were removed, debridement done, bone fragments were docked followed by application of ring fixator and compression. In aseptic nonunion (*n*=13), distraction for three weeks followed by compression was the protocol. Early shoulder and elbow physiotherapy was instituted. The apparatus was removed after clinical and radiological union and the results were assessed for bone healing and functional status.

**Results:**

Fracture union was achieved in all the 19 cases. Pin site infection was seen in 2 cases (10.52%). The bone healing results were excellent in eighteen cases (94.73%) and good in one case (5.26%).The functional results were found to be excellent in fourteen cases (73.68%), good in four (21.05%) and fair in one case (5.26%).

**Conclusion:**

Ilizarov’s method is an excellent option for treatment of septic and aseptic non union of diaphyseal fractures of the humerus as it addresses all the problems associated with non union of the humerus like infection, deformity and joint stiffness.

## INTRODUCTION

Fractures of the humerus constitute about 3% of all fractures.^1^ They are usually treated by either conservative methods or by operative methods.^1^ Nonunion can occur after both modalities of treatment.^2^–^6^ The treatment of nonunion presents a difficult proposition to both the patient and the treating surgeon. The presence of infection as usually seen in nonunion after primary surgical management further compounds the problem.^7^,^8^ The common modalities of treatment of aseptic nonunion are plating and cancellous bone grafting, intramedullary nailing and bone grafting and Ilizarov’s method of external ring fixation. In infected nonunion, the usual treatment protocol is removal of hardware, if any, debridement and some form of external fixation followed by bone grafting once the infection subsides.^7^–^9^ The advantage of Ilizarov’s method is that it can be done even in the presence of infection and the deformity, if any, can also be corrected simultaneously. There is no necessity for bone grafting in most cases.^5^,^10^,^11^ This prospective study discusses the outcome of nonunion of diaphyseal fractures of the humerus treated by Ilizarov’s method.

## MATERIALS AND METHODS

Nineteen patients with diaphyseal fractures of the humerus with an age range of 18 to 57 years were included in the study. There were thirteen males and six females. The mean period of nonunion was 10.4 months (range nine to fourteen months).The initial fracture was open in 9 cases. The primary management was plates and screws (*n*=11), intramedullary nailing (*n*=1) and conservative methods (*n*=7). There were six infected nonunions. 12 had undergone mean 1.7 surgeries (1-3 previous surgeries). The cases were carefully assessed both clinically and radiologically preoperatively. The associated problems like infection, deformity and joint stiffness were documented. Preoperative shoulder stiffness was found in fifteen cases and elbow stiffness in six cases. Nonunion was seen at the middle third of the humerus (*n*=12), at the junction of upper and middle third (*n*=5) and in the lower third (*n*=2). Ring fixator using the principles of Ilizarov was applied either by free hand technique (*n*=15) or using a pre assembled construct (*n*=4). The apparatus was assembled with the plan of correcting all the 3 dimensional deformities and achieve bony union. Two levels of fixation were done in each segment of bone. In the proximal fragment, fixation was done using either Italian arches or Omega arches and 5/8^th^ rings and Schanz pins. The distal segment was fixed using either - one full ring and a 5/8^th^ ring or a full ring and a drop wire depending on the length of the fragment. In post surgical infected non union (*n*=6), the implants were removed after isolating the radial nerve. After thorough debridement, the bone fragments were docked and ring fixator was applied followed by compression. In post surgical aseptic non union (*n*=6), the implants were removed after isolation of the radial nerve, the intervening fibrous tissue was excised followed by application of ring fixator. Inj. Ceftriaxone 1g i.v b.i.d was given in the post operative period for 3 days followed by antibiotics based on the culture and sensitivity of the material obtained after debridement for a period of three weeks. The most commonly used antibiotic was Ofloxacin 400 mg bid. In aseptic nonunion, following conservative treatment, initial distraction was done for three weeks at the rate of 1 mm/ day to break the fibrous tissue present at the non union site, followed by compression. In six cases 2 K wires in the form of intramedullary pins were inserted when the bone ends were tapered and translation was expected on docking and where excision of the tapered ends would have caused unacceptable shortening. Compression was done at the rate of 1 mm/day in two increments of 0.5 mm/ 12 hours till the patient felt pain at the docking site. Thereafter compression was continued at the rate of 0.25 mm every 3 days (i.e. 1 mm in 12 days). Bone grafting was not done in any case. Postoperatively, shoulder and elbow exercises were encouraged and slowly progressed to activities of daily living. The patients were instructed to frequently clean the pin sites. The patients were followed up every month until union followed by three monthly visits. The apparatus was removed after clinical and radiological union achieved. Abnormal mobility was assessed before removal of the apparatus by disconnecting the rings on either side of the nonunion site and adding angulatory force to elicit any deformation.

The final outcome of treatment was judged by both union and functional status of the limb. The bone healing was graded, according to Paley’s criteria,^12^ as excellent when union was achieved along with absence of infection, a deformity<7° and limb length discrepancy<2.5 cm. It was graded as good when there was union along with any two of the other three criteria and fair when only one of the three criteria was fulfilled along with union. Nonunion along with a persistent or recurrent infection was considered a poor result. Paley used these criteria for tibial defects but no criteria is available for upper limb defects to the best of our knowledge.

The final functional result^13^ was graded as excellent when there was shoulder abduction>150°, no loss of >10° of movement in any direction, full strength at elbow and shoulder joints and absence of pain at the non union site and adjacent joints. It was graded as good when shoulder abduction>120°, no loss of >15° of motion in any direction, full strength at adjacent joints and absence of pain. A fair result was shoulder abduction 90°-120°, no loss of movement>20° in any direction, less than full strength in elbow and shoulder with mild manageable pain. Shoulder abduction<90°, loss of motion>20°, gross decrease in power in shoulder and elbow with pain hampering activities of daily living was considered a poor result. The patients were followed up for a period of 14 to 38 months (mean 27 months).

## RESULTS

**Figure 1:**
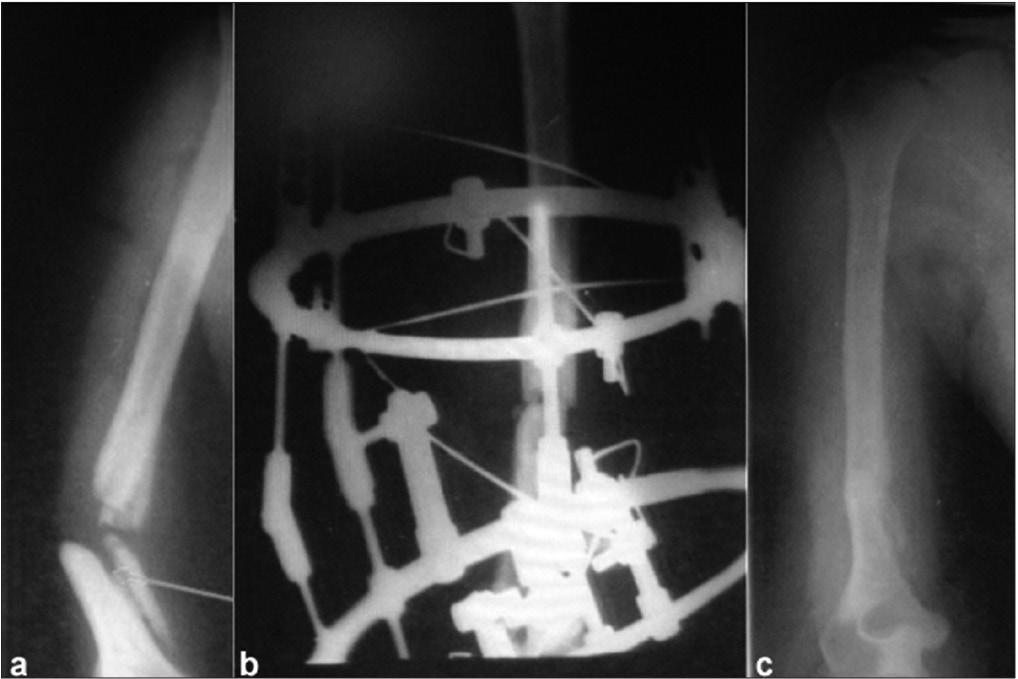
Anteroposterior radiograph of a 31 year old patient showing (a) 9 months old aseptic nonunion after conservative treatment (b) ring fixator *in situ* (c) Radiological union at 24 weeks

**Figure 2:**
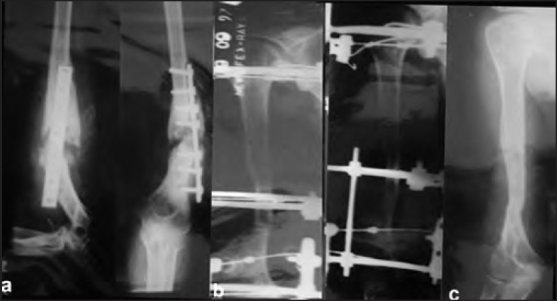
(a) Lateral and anteroposterior radiograph showing 10 month old post surgical infected nonunion (b) radiograph (lateral and anteroposterior view) showing ring fixator *in situ* (c) radiograph (anteroposterior view) showing radiological union at 26 weeks

**Figure 3:**
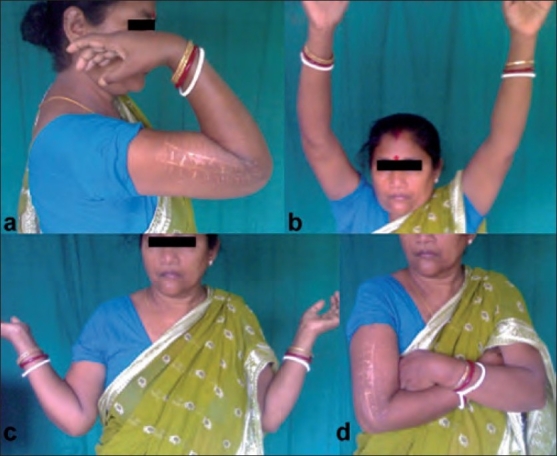
Clinical photograph of a patient treated with Illizarov fixator showing (a) elbow flexion (b) shoulder abduction (c) shoulder external rotation (d) shoulder internal rotation

Union was achieved in all 19 cases within a mean period of 6.4 months (six to nine months) [Figures 1 and 2]. Pin site infection was seen in two cases (10.52%). They were superficial and were successfully treated by local cleansing and antibiotics. There was one case of radial nerve palsy seen in a patient where plate removal was done which recovered completely in three months time. Infection at the non union site subsided in all cases. Shortening of 3 cm was observed in one case (5.26%). In all other cases it was less than 2.5 cm. The bone healing results were excellent in eighteen cases (94.73%) and good in one case (5.26%).The functional results were found to be excellent in fourteen cases (73.68%), good in four cases (21.05%) and fair in one case(5.26%) [Figure 3] [Table 1].

**Table 1:** Clinical details of the patients

No.	Sex	Time since fracture (months)	Fracture type	Time to union (in weeks)	Infection	Final LLD (cm)	Final angulation in any plane	Shoulder abduction	Pain
1	M	9	Closed	25	No	0	4°	150°	No
2	M	11	Open	26	No	2.1	5°	165°	No
3	M	9	Closed	24	No	0	3°	170°	No
4	F	10	Closed	26	No	0	0°	160°	No
5	M	14	Open	25	No	1.8	5°	130°	No
6	F	11	Open	26	No	1.6	2°	155°	No
7	M	12	Closed	25	No	0	4°	150°	No
8	M	9	Closed	24	No	0	2°	160°	No
9	F	10	Open	36	No	3	6°	100°	No
10	M	13	Open	27	No	2.1	6°	135°	No
11	M	9	Open	24	No	0	0°	170°	No
12	F	10	Closed	24	No	0	5°	160°	No
13	M	9	Closed	24	No	0	0°	160°	No
14	F	10	Open	26	No	1.8	4°	130°	No
15	M	10	Closed	26	No	1.2	5°	155°	No
16	M	9	Closed	24	No	0.8	2°	160°	No
17	M	12	Open	27	No	1.2	5°	155°	No
18	M	10	Closed	24	No	0	5°	150°	No
19	F	12	Open	25	No	2.2	6°	140°	No

M=Male, F=Female, LLD=Limb Length Discrepancy

## DISCUSSION

Most diaphyseal fractures of the humerus can be treated nonoperatively. Nonunion occurs in approximately 10% of patients with humeral shaft fractures regardless of the type of treatment i.e. both conservative and surgical methods.^2^,^3^,^4^,^6^,^10^,^11^ The common causes of nonunion are infection, distraction at fracture site, unstable fixation, wrong choice of implant, iatrogenic devitalization of soft tissues, bone loss and osteoporosis.^14^ Non unions can be hypertrophic or atrophic with or without infection. Infection is usually seen in post surgical cases and open fractures. Hypertrophic nonunion of humerus is usually due to improper and inadequate stabilization and depicts a mechanical failure.^16^ Atrophic nonunion is a biological failure to unite.^16^ Debridement and freshening of bone ends along with sequestrectomy, if sequestrum is present, is indicated in cases of gross infection. Exchange nailing 4,15 for nonunion following intramedullary nailing has not been as successful as in other long bones like femur or tibia. Ilizarov’s ring fixator 5,6,8,10,11,14,16 is useful, especially in such infected cases where plating and bone grafting would result in persistence of infection and non union.^4^ Adherence to the safe zones during pin insertion prevents any neurovascular complications. Application of the ring fixator enables correction of the incumbent deformity either intraoperatively or gradually postoperatively. Minimal residual deformity in the humerus is functionally insignificant.

The rationale behind our protocol is that after distraction, initial compression at the rate of 1mm/day is done to bring the bone segments into maximal compression indicated by the appearance of pain. Further compression at the same rate would cause intense pain and bending of the wires in the construct. Thus the compression rate is slowed down to 1mm/12 days.^13^ The time taken for union in our series (mean 6.4 months) is comparable with other series in literature like Lammens *et al*.^5^ (mean 4.5 months), Cattaneo *et al*.^18^ (mean 7.5 months) and Bari *et al*.^17^ (mean 8 months).The success rate in our series (100%) is good in comparison to other series like Cattaneo *et al*.^18^ (86%), Lammens *et al*.^5^ (93%) and Maini *et al*.^8^ (90%). Pin site infection is the most common complication in our series (10.52%) as in other series in literature.^5^,^8^,^11^ Other complications like persistence of infection, refracture and vascular injury were not encountered.^5^,^8^,^11^ The bone healing and functional results (excellent in 94.73% and 73.68% respectively) achieved also compared well with other series.^8^,^11^,^16^ The rigid fixation achieved enables early institution of shoulder and elbow physiotherapy, thus improving the mobility of the adjacent joints and reducing stiffness and return to activities of daily living along with excellent results.^5^,^11^,^15^

## CONCLUSION

Ilizarov’s method addresses all the problems associated with nonunion of the humerus like infection, deformity and joint stiffness. It gives good to excellent functional results. Thus Ilizarov’s method of ring fixation is an excellent modality of treatment of septic and aseptic nonunion of diaphyseal fractures of the humerus.
